# Supplementary material for: Spectroscopy of corannulene cations in helium nanodroplets
Source: Faraday Discuss. 2018 Dec 21;217(0):276–89. doi: 10.1039/c8fd00178b (PMC6677026; doi:10.1039/c8fd00178b)
Supplement: Supplementary file 1 [file FD-217-C8FD00178B-s001.pdf]

Electronic Supplementary Material (ESI) for Faraday Discussions.
This journal is © The Royal Society of Chemistry 2019

Electronic Supplementary Information: Spectroscopy of corannulene cations in helium nanodroplets

Michael Gatchell,[a,b]* Paul Martini,[a] Felix Laimer,[a] Marcelo Goulart,[a] Florent Calvo,[c] and Paul Scheier[a]

[a]Institut für Ionenphysik und Angewandte Physik, Universität Innsbruck, Technikerstr. 25, A-6020 Innsbruck, Austria
[b]Department of Physics, Stockholm University, 106 91 Stockholm, Sweden.
[c]Université Grenoble Alpes, CNRS, LIPhy, Grenoble, France

April 12, 2019

Structure of corannulene cation

Below we give the Cartesian coordinates and partial atomic charges carried by the hydrogen and carbon atoms of the corannulene molecular ion, as employed in our atomistic simulations. All coordinates are given in Å.

Atom type	x	y	z	q
C	0.949718	0.719485	0.598133	0.063726
C	0.949718	-0.719485	0.598133	0.063726
C	-0.367301	-1.156607	0.639582	-0.101897
C	-1.198325	0.000000	0.625876	0.235755
C	-0.367301	1.156607	0.639582	-0.101897
C	1.991511	1.479154	0.074670	0.188579
C	1.991511	-1.479154	0.074670	0.188579
C	-0.755876	-2.371747	0.102386	0.187068
C	-2.448704	0.000000	0.064132	0.107907
C	-0.755876	2.371747	0.102386	0.187068
C	0.331397	3.269232	-0.227435	-0.214911
C	1.640354	2.849996	-0.240500	-0.222880
C	3.117124	0.717081	-0.294847	-0.192637
C	3.117124	-0.717081	-0.294847	-0.192637
C	1.640354	-2.849996	-0.240500	-0.222880
C	0.331397	-3.269232	-0.227435	-0.214911
C	-2.139378	-2.438061	-0.269943	-0.164007
C	-2.944386	-1.310856	-0.287206	-0.186195
C	-2.944386	1.310856	-0.287206	-0.186195
C	-2.139378	2.438061	-0.269943	-0.164007
H	0.105455	4.276357	-0.561363	0.195868
H	2.409756	3.536676	-0.576219	0.192555
H	3.993091	1.207186	-0.708720	0.199349
H	3.993091	-1.207186	-0.708720	0.199349
H	2.409756	-3.536676	-0.576219	0.192555
H	0.105455	-4.276357	-0.561363	0.195868

*michael.gatchell@uibk.ac.at

H	-2.551224	-3.369648	-0.643383	0.190782
H	-3.956728	-1.412285	-0.665359	0.192769
H	-3.956728	1.412285	-0.665359	0.192769
H	-2.551224	3.369648	-0.643383	0.190782

Band positions as functions of solvation

The band positions are show as a function of the number of solvating He atoms (n). All values of n are not measurable for every band and some gaps are therefore present.

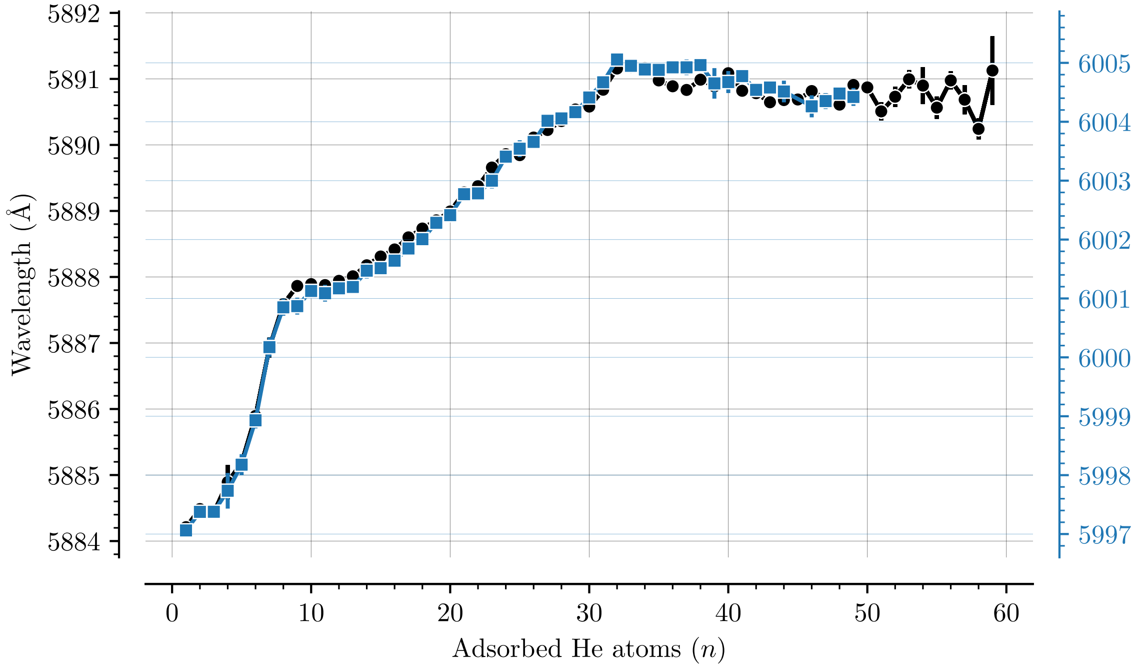

Figure 1: Position of band A (blue squares, right scale) versus band D (black circles, left scale).

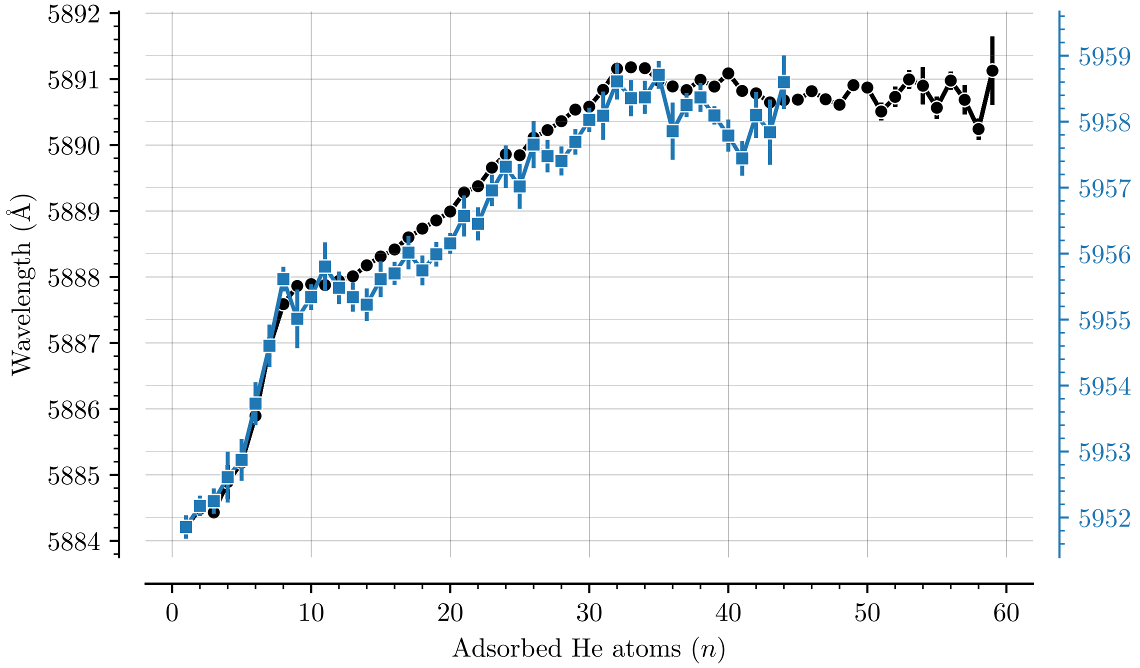

Figure 2: Position of band B (blue squares, right scale) versus band D (black circles, left scale).

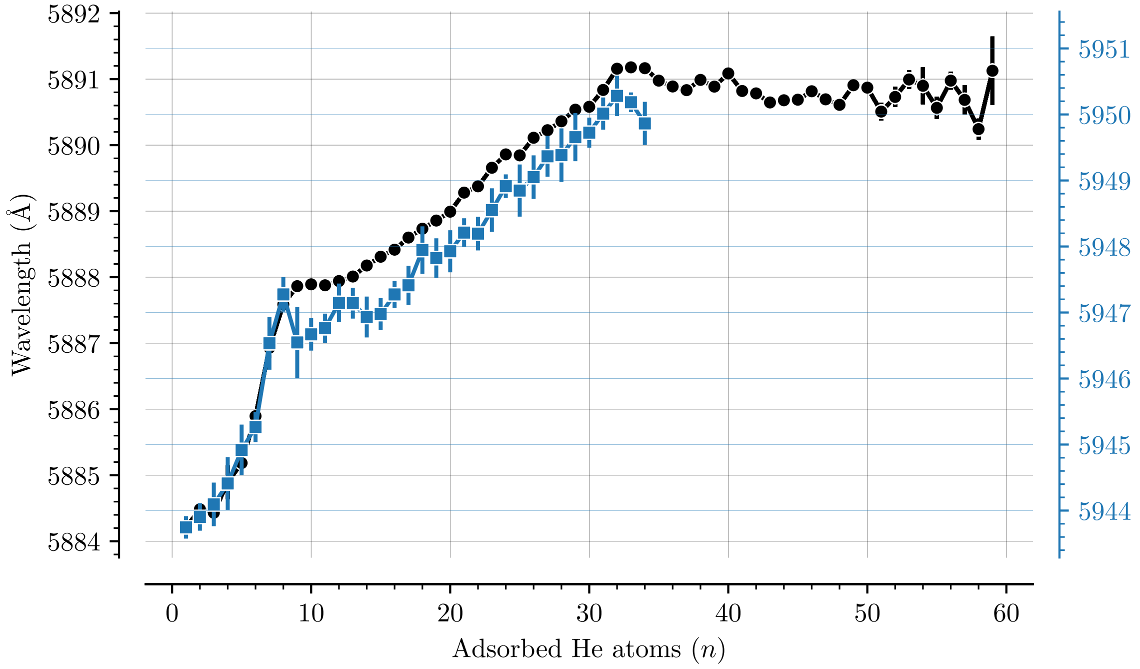

Figure 3: Position of band C (blue squares, right scale) versus band D (black circles, left scale).

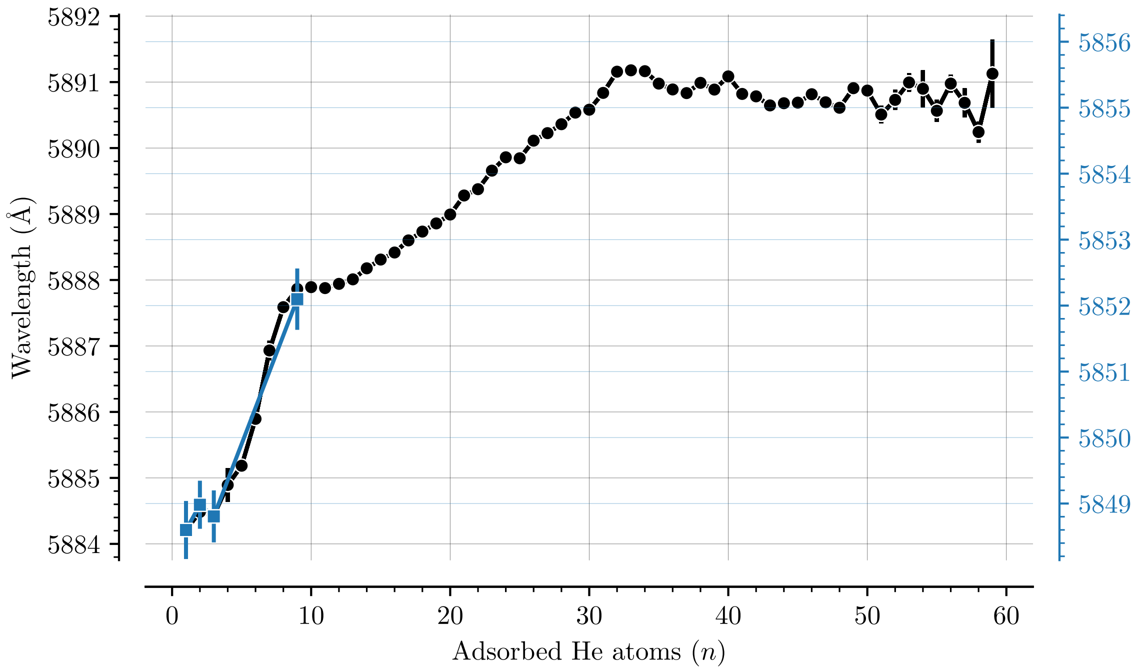

Figure 4: Position of band E (blue squares, right scale) versus band D (black circles, left scale).

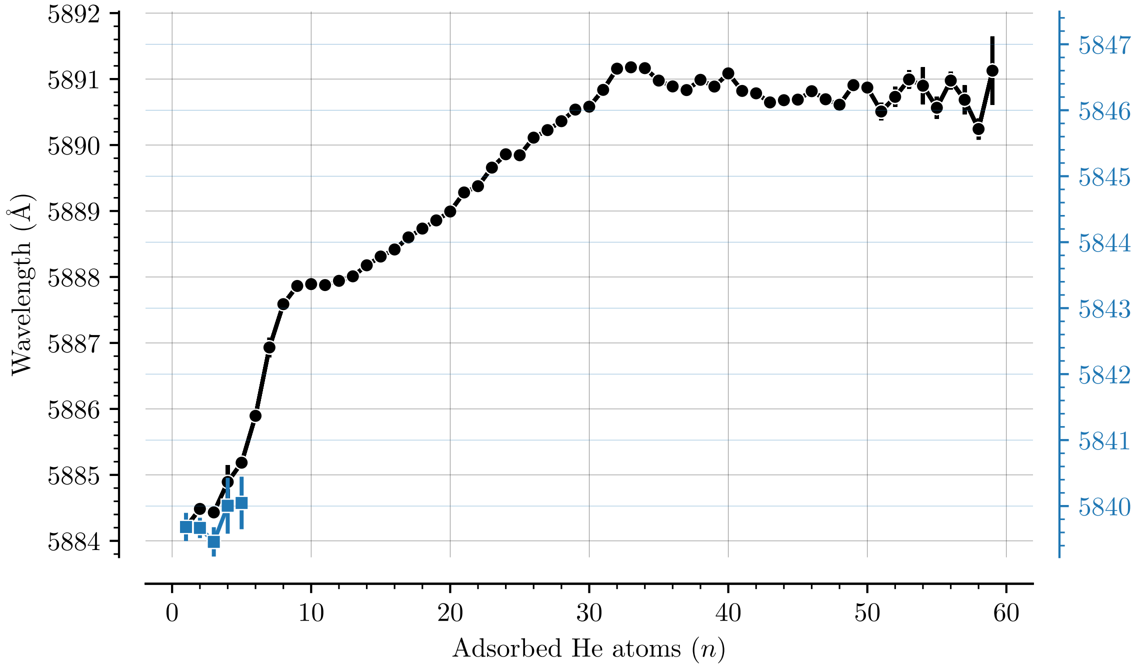

Figure 5: Position of band F (blue squares, right scale) versus band D (black circles, left scale).

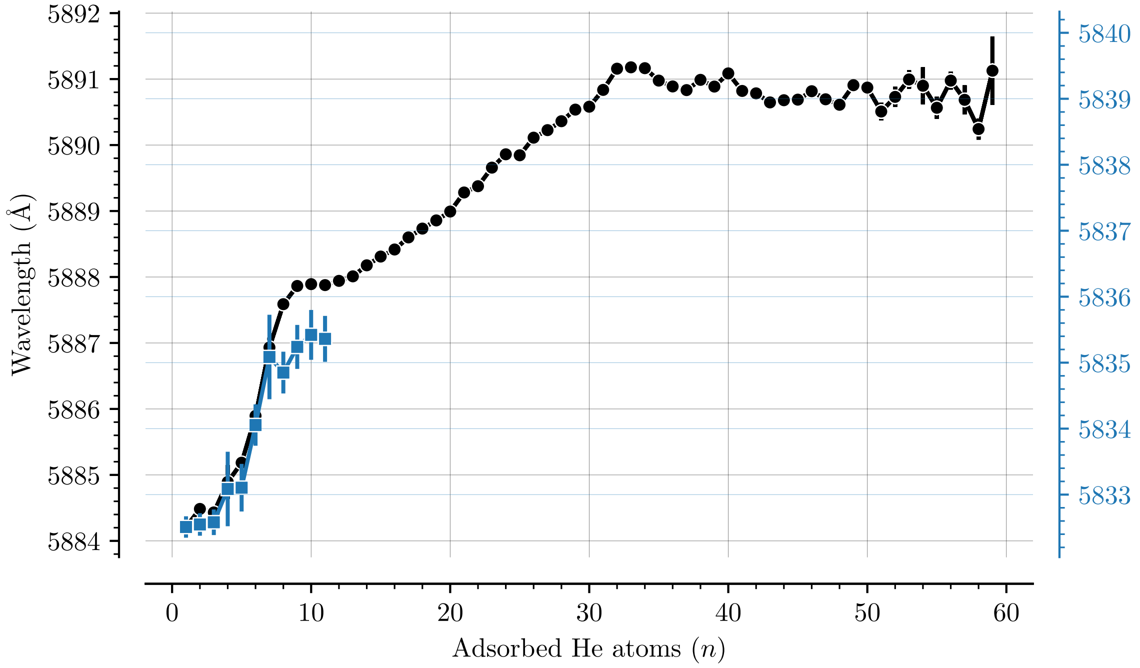

Figure 6: Position of band G (blue squares, right scale) versus band D (black circles, left scale).

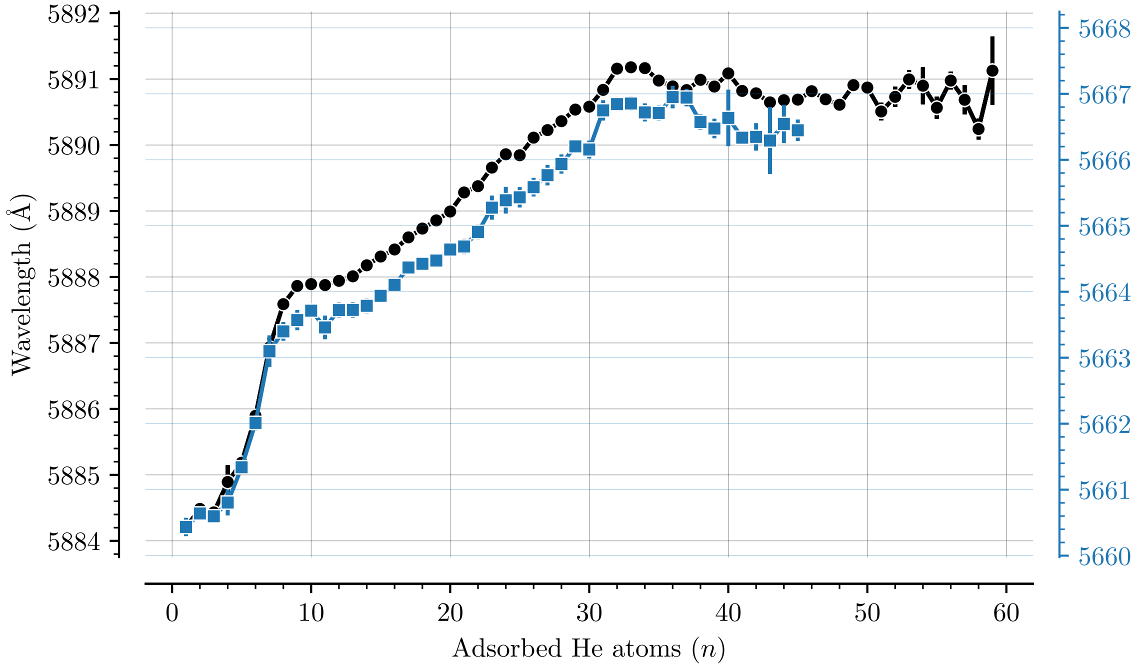

Figure 7: Position of band I (blue squares, right scale) versus band D (black circles, left scale).

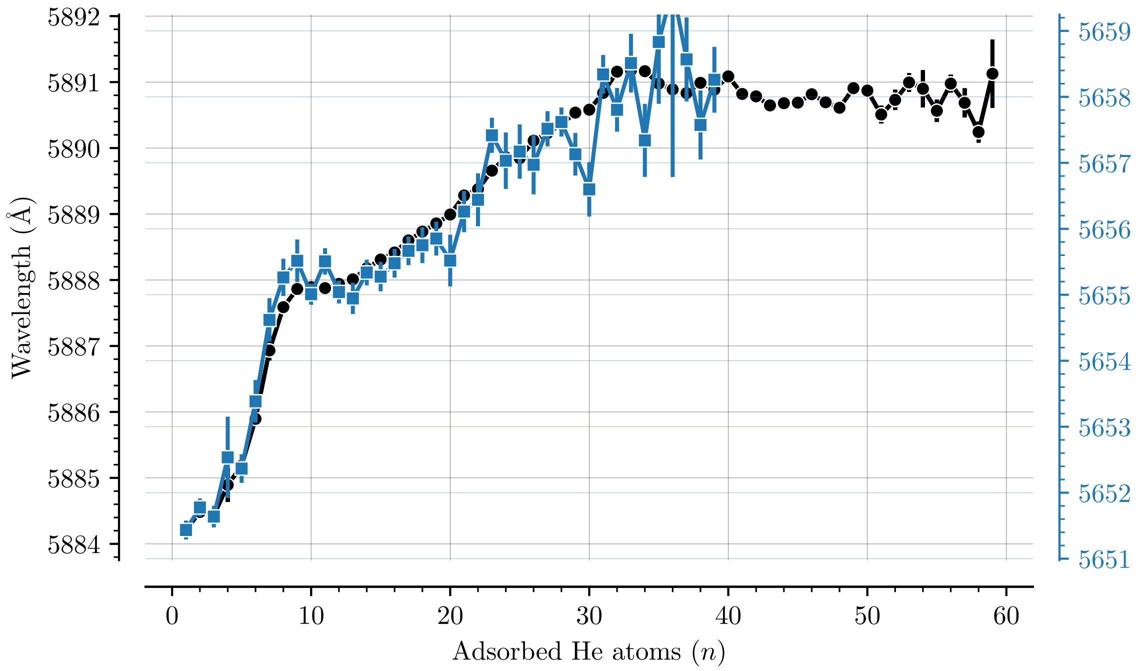

Figure 8: Position of band J (blue squares, right scale) versus band D (black circles, left scale).

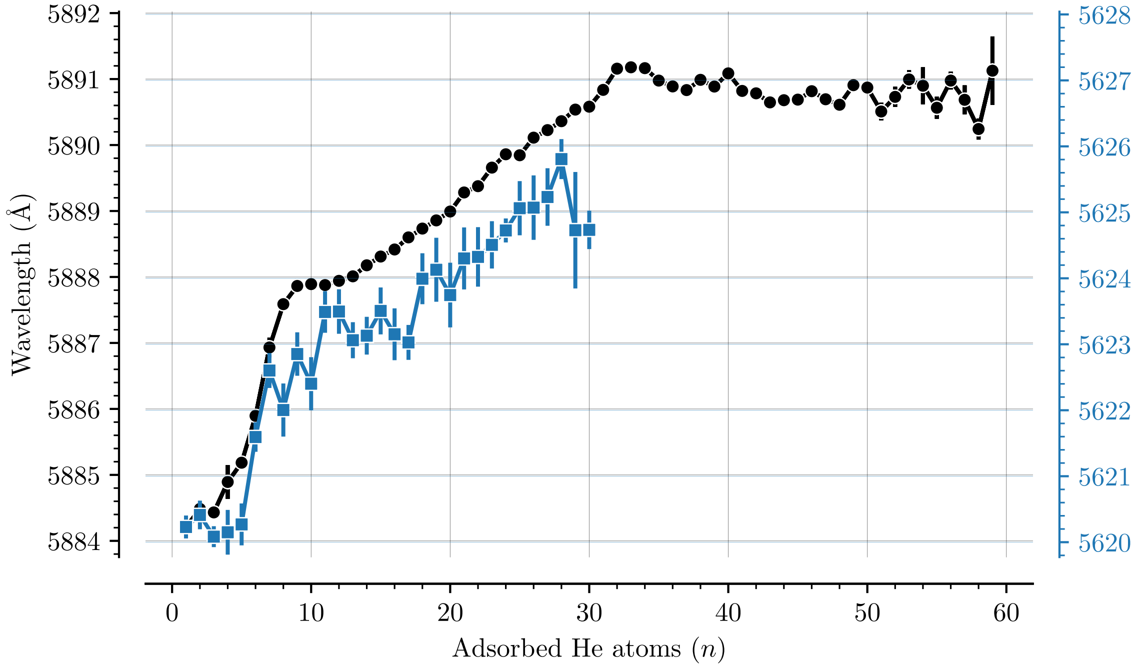

Figure 9: Position of band K (blue squares, right scale) versus band D (black circles, left scale).

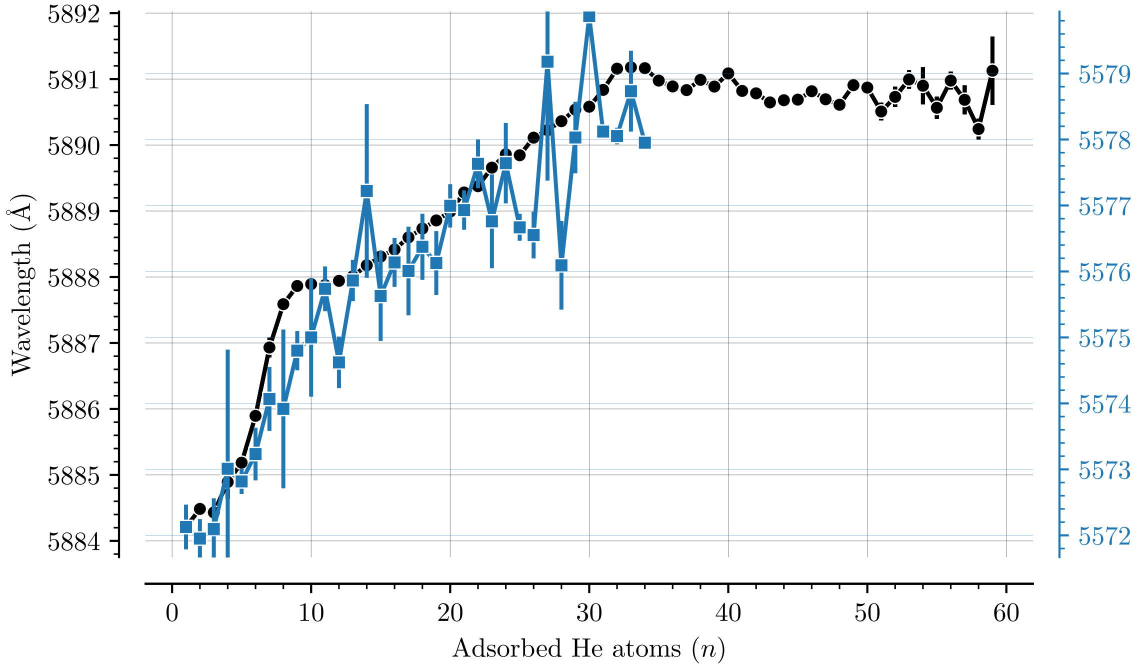

Figure 10: Position of band L (blue squares, right scale) versus band D (black circles, left scale).

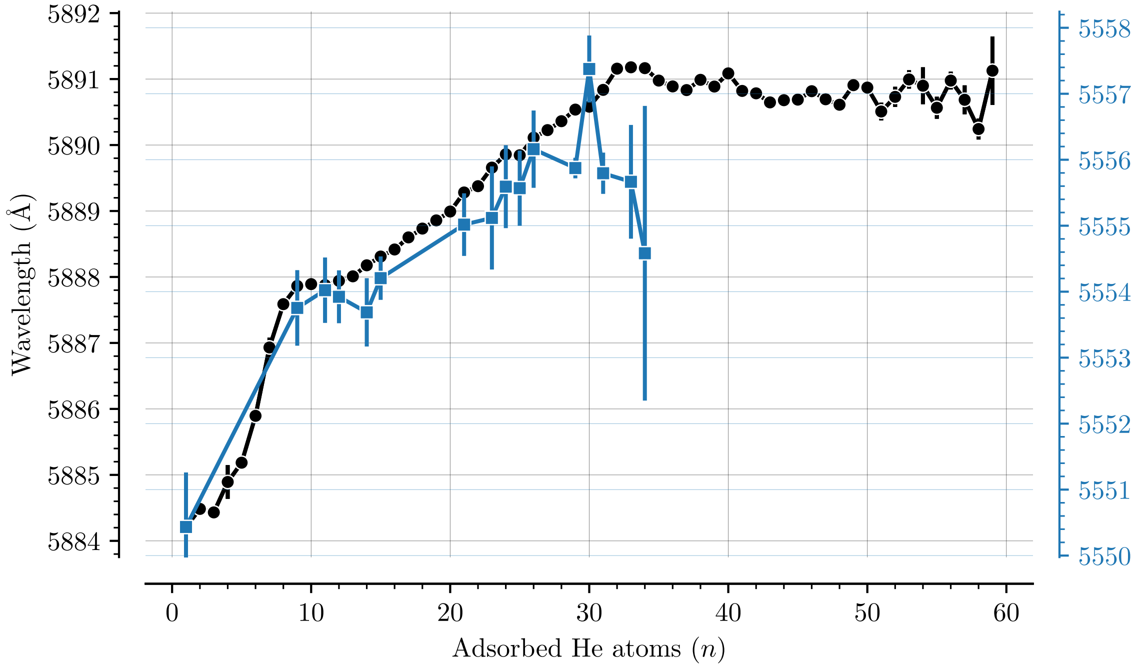

Figure 11: Position of band M (blue squares, right scale) versus band D (black circles, left scale).
